# Adverse events related to neuromuscular blocking agents: a disproportionality analysis of the FDA adverse event reporting system

**DOI:** 10.3389/fphar.2024.1403988

**Published:** 2024-07-24

**Authors:** Liangxia Li, Qianqian Xu, Yarui Liu, Liangfang Pang, Zhou Cui, Yuanyuan Lu

**Affiliations:** Department of Pharmacy, Maternal and Child Health Hospital of Hubei Province, Tongji Medical College, Huazhong University of Science and Technology, Wuhan, China

**Keywords:** neuromuscular blocking agents, adverse events, FAERS, disproportionality analysis, safety

## Abstract

**Background:** Neuromuscular blocking agents (NMBAs) are primarily used during surgical procedures to facilitate endotracheal intubation and optimize surgical conditions. This study aimed to explore the adverse event signals of NMBAs, providing reference for clinical safety.

**Methods:** This study collected reports of atracurium, cisatracurium, rocuronium, and vecuronium as primary suspect drugs in The US Food and Drug Administration Adverse Event Reporting System (FAERS) from the first quarter of 2004 to the third quarter of 2023. The adverse events (AEs) reported in the study were retrieved based on the Preferred Terms (PTs) of the Medical Dictionary for Regulatory Activities. In addition, we conducted disproportionality analysis on relevant reports using the reporting odds ratio (ROR) method and Bayesian confidence propagation neural network (BCPNN) method. A positive signal was generated when both algorithms show an association between the target drug and the AE.

**Results:** A total of 11,518 NMBA-related AEs were reported in the FAERS database. The most AEs of rocuronium were collected. NMBA-related AEs involved 27 different system organs (SOCs), all of the four NMBAs had positive signals in “cardiac disorders,” “immune system disorders,” “respiratory, thoracic and mediastinal disorders” and “vascular disorders.” At the PTs level, a total of 523 effective AEs signals were obtained for the four NMBAs. AEs labled in the instructions such as anaphylaxis (include anaphylactic reaction and anaphylactic shock), bronchospasm, respiratory arrest and hypotension were detected positive signals among all NMBAs. In addition, we also found some new AEs, such as ventricular fibrillation for the four NMBAs, hyperglycaemia for atracurium, kounis syndrome and stress cardiomyopathy for rocuronium, hepatocellular injury for cisatracurium, hyperkalaemia for vecuronium. To further investigated the AEs associated with serious clinical outcomes, we found that cardiac arrest and anaphylaxis were the important risk factors for death due to NMBAs.

**Conclusion:** NMBA-related AEs have a significant potential to cause clinically severe consequences. Our study provides valuable references for the safety profile of NMBAs, and considering the limitations of the FAERS database, further clinical data are needed to validate the findings of this study.

## Introduction

Neuromuscular blocking agents (NMBAs) are a class of medications commonly used in anesthesia (McLean et al., 2015).They are used to facilitate airway management, improve surgical conditions, and insure immobility during critical points in an operation (Thilen et al., 2023). The NMBAs are administered over 100 million times annually to facilitate tracheal intubation (Macartney, 2013). Meta-analysis of clinical trials had shown that avoiding NMBAs during endotracheal intubation significantly increased the incidence of difficult intubation and discomfort (Lundstrøm et al., 2018). NMBAs are primarily categorized into two types: depolarizing NMBAs (e.g., succinylcholine) and non-depolarizing NMBAs (e.g., atracurium, cisatracurium, rocuronium, vecuronium). The ideal NMBA is considered to be a non-depolarizing agent that has a rapid onset of action, short duration of effect, and minimal side effects (Macartney, 2013). Atracurium, cisatracurium, rocuronium and vecuronium are frequently used non-depolarizing NMBAs in clinical practice. They act by competitively blocking acetylcholine receptors at the neuromuscular junction, producing prolonged muscle relaxation (Naguib et al., 2002). Cisatracurium is the stereoisomer of atracurium, and both belong to the benzylisoquinoline NMBAs. Compared to atracurium, cisatracurium has four times the potency on neuromuscular blockade (Kim et al., 2016). Atracurium and cisatracurium are the preferred drugs for continuous infusion. The latter, cisatracurium, is the NMBA of choice in the treatment of critically ill patients requiring neuromuscular blockade as it is unrelated to histamine release (Tsolaki et al., 2022). Rocuronium and vecuronium are both steroid NMBAs. Rocuronium has a rapid onset and intermediate duration of action, making it suitable for rapid sequence intubation (Tsolaki et al., 2022). In comparison, vecuronium has a slower onset but a similar duration of action to rocunorium (Engbaek et al., 1994).

The widespread use of NMBAs has raised concerns among clinicians and researchers regarding their related safety issues. During the process of general anesthesia, drug-related adverse events (AEs) can be severe and life-threatening, with NMBAs being the primary cause of these adverse events (Di Leo et al., 2018). Common AEs include anaphylaxis, hypotension, bronchospasm and bradycardia (Jick et al., 1989; Arnot-Smith et al., 2010; Petitpain et al., 2018). However, most of the evidence for NMBA-associated AEs came from case reports and clinical trials, with relatively small sample sizes. It is currently unclear whether NMBAs may cause other serious AEs. Thus, conducting comprehensive and large-scale study on AEs related to NMBAs is crucial. In this study, we collected AEs of NMBAs from the FDA Adverse Event Reporting System (FAERS), and assessed the potential relevance between NMBAs and AEs through risk factor analysis. The aim of this study is to investigate the overall safety profile of NMBAs and provide a reference for further identification of NMBA-related adverse events.

## Materials and methods

### Data source and preprocessing

FAERS is a comprehensive and publicly available database that collects and stores information on adverse events and medication errors reported to the FDA. It serves as a valuable resource for monitoring the safety of pharmaceutical products, identifying potential risks, and facilitating post-marketing surveillance. AEs reported in FAERS include any untoward medical occurrence associated with the use of a medication, including both known and unknown side effects.

The FAERS database is updated quarterly. Each FAERS data package consists of several different files: Patient Demographic Information (DEMO), Drug Information (DRUG), adverse events (REAC), Patient outcomes (OUTC), Report Source information (RPSR), Drug Therapy Start and End Date (THER), and Drug indication information (INDI). Additionally, starting from the first quarter of 2019, DELETED data sheets were added to include revoked or withdrawn case data. We downloaded all reports from the first quarter of 2004 to the third quarter of 2023. Using Structured Query Language (SQL), we extracted and analyzed all reported AEs related to the targeted NMBAs (atracurium, cisatracurium, rocuronium and vecuronium). In order to enhance the accuracy and credibility of the results, we screened the role_code as “PS” (primary suspected) in the DRUG files, removed duplicate reports and those in the DELETED data sheets, and the remaining reports were included in the study, which we referred to as valid reports. Each report represents a unique patient, and it is important to note that a single patient may experience and report several different AEs. According to the FDA recommendations, we utilized MySQL 8.0 to correlate these subsets of the database and remove duplicate information. The deduplication process was performed based on the following criteria: when the CASEID (number for identifying a FAERS case) was the same, we selected the latest FDA_DT (the date when the FDA received the case), and when CASEID and FDA_DT were the same, we chose the record with the higher PRIMARYID (unique identifier for the FAERS report)

Due to the diverse backgrounds of reporters, the drug names and AEs names reported in the FAERS database are often inconsistent and non-standardized. To identify all the records of target drugs in FAERS, we extracted reports using the brand names, generic names, and chemical names of the target NMBA. Medical Dictionary for Regulatory Activities (MedDRA) is a standardized medical terminology that developed for recording and reporting of AE data (Brown, 2004; Singh, 2015). Its hierarchical structure consists of five levels, ranging from the lowest level terms (LLT) to system organ class (SOC). SOC is the highest level of terminology used for classifying AEs. The AEs in our collection were coded using the preferred term (PT) and then mapped to their corresponding SOC level in MedDRA (version 26.1). Serious clinical outcomes were classified as death, disability, hospitalization (initial or prolonged), life-threatening complications, required intervention to prevent permanent impairment or damage and other serious medical events.

### Disproportionality analysis

In recent years, disproportionality analysis (Fusaroli et al., 2024) has emerged as an important approach for evaluating drug safety. To comprehensively assess the safety profile of the NMBAs, we conducted disproportionality analysis at both the SOC level and the PT level. The analysis at SOC level helps identify the potential association between NMBAs and specific systems or organs, providing a broader and more macro-level perspective. While the analysis at PT level is more specific, focusing on particular adverse event terms. It aids in discovering new adverse event signals and provides more detailed and specific information. Combining both analyses can lead to a comprehensive and systematic discovery of signals.

Two algorithms, including the reporting odds ratio (ROR) and Bayesian confidence propagation neural network (BCPNN), were used to detect whether NMBAs were significantly associated with AEs (Zhang et al., 2022; Wang et al., 2023). Because the number of AEs for a specific PT less than 3 cases may lead to false positive signals, they were not included in the analysis (Zhou et al., 2022). To further avoid false positive signals, a positive signal was generated only when both algorithms met the criteria. In the formulas below, the value a represents the number of reports that contain both the target drug and the target adverse event, b represents the number of reports that contain the target drug with other adverse events, c represents the number of reports that contain the target adverse event related to other drugs, d represents the number of reports that contain other drugs and other adverse events (Zhang et al., 2022). The specific formulae are as follows:

### ROR

$$\begin{array}{l} ROR=\frac{ad}{bc}\\ 95\%CI=e^{In\left(ROR\right)\pm 1.96\sqrt{\frac{1}{a}+\frac{1}{b}+\frac{1}{c}+\frac{1}{d}}} \end{array}$$

If the lower limit of 95% CI > 1 and a ≥ 3, it should be considered a positive signal.

### BCPNN

$$\begin{array}{l} IC=\log_{2}\frac{a\left(a+b+c+d\right)}{\left(a+b\right)\left(a+c\right)}\\ IC-2SD=E\left(IC\right)-2\sqrt{V\left(IC\right)} \end{array}$$

If IC-2SD > 0, it should be defined a positive signal; Specifically, if 0 < IC- 2SD ≤ 1.5, it should be defined as a weak signal (+); if 1.5 < IC- 2SD ≤ 3, it should be defined as a medium signal (++); if IC-2SD > 3, it should be defined as a strong signal (+++).

## Results

### Descriptive analysis

During the study period from January 2004 to September 2023, FAERS received a total of 16, 961, 231 valid reports, among which 4,619 reports were identified with NMBAs as the primary suspected drug. The percentage of reports was highest for rocuronium (3,167 reports, 68.56%), followed by atracurium (506 reports, 10.95%), vecuronium (474 reports, 10.26%), and cisatracurium (472 reports, 10.22%). The clinical characteristics of the patients are summarized in Table 1.

**TABLE 1:** The characteristics of the reports related to NMBAs from January 2004 to September 2023.

	Atracurium (N^a^, %)	Cisatracurium (N^a^, %)	Rocuronium (N^a^, %)	Vecuronium (N^a^, %)	Total (N^a^, %)
Gender					
Female	267 (52.77)	167 (35.38)	1,323 (41.77)	174 (36.71)	1931 (41.81)
Male	213 (42.09)	203 (43.01)	1,233 (38.93)	179 (37.76)	1828 (39.58)
Unknown	26 (5.14)	102 (21.61)	611 (19.29)	121 (25.53)	860 (18.62)
Age (year)^b^					
0–17	51 (10.08)	24 (5.08)	228 (7.20)	73 (15.40)	376 (8.14)
18–59	240 (47.43)	175 (37.08)	1,182 (37.32)	148 (31.22)	1745 (37.38)
60–74	115 (22.73)	100 (21.19)	647 (20.43)	65 (13.71)	927 (20.07)
75–89	29 (5.73)	29 (6.14)	263 (8.30)	33 (6.96)	354 (7.66)
≥90	1 (0.20)	2 (0.42)	14 (0.44)	1 (0.21)	18 (0.39)
Unknown	70 (13.83)	142 (30.08)	833 (26.3)	154 (32.49)	1,199 (25.96)
Reporter Country					
China	21 (4.15)	74 (15.68)	13 (0.41)	3 (0.63)	111 (2.40)
France	241 (47.63)	116 (24.58)	298 (9.41)	11 (2.32)	666 (14.42)
Great Britain	153 (30.24)	9 (1.91)	334 (10.55)	22 (4.64)	518 (11.21)
Japan	1 (0.20)	2 (0.42)	299 (9.44)	108 (22.78)	410 (8.88)
United States	18 (3.56)	164 (34.75)	1,097 (34.64)	191 (40.3)	1,470 (31.83)
Other countries	72 (14.23)	107 (22.67)	1,126 (35.55)	139 (29.32)	1,444 (31.26)
Reported person					
Consumer	64 (12.65)	8 (1.69)	347 (10.96)	17 (3.59)	436 (9.44)
Health professional	19 (3.75)	47 (9.96)	277 (8.75)	28 (5.91)	371 (8.03)
Lawyer	0 (0)	0 (0)	3 (0.09)	0 (0)	3 (0.06)
Physician	203 (40.12)	190 (40.25)	935 (29.52)	78 (16.46)	1,406 (30.44)
Pharmacist	42 (8.30)	107 (22.67)	636 (20.08)	120 (25.32)	905 (19.59)
Other health professional	142 (28.06)	79 (16.74)	772 (24.38)	96 (20.25)	1,089 (23.58)
Unknown	36 (7.11)	41 (8.69)	197 (6.22)	135 (28.48)	409 (8.85)

^a^ Number of reports.

^b^ The patients were grouped according to the age classification criteria of the United Nations World Health Organization: children under 18 years old, young adults 18–59 years old, pre-geriatric age 60–74 years old, mid-geriatric age 74–89 years old, and terminal age 90^+^ years old.

NMBAs, Neuromuscular blocking agents.

The incidence of atracurium-related AEs appeared to be higher in females compared to males (52.77% vs. 42.09%). For cisatracurium, 21.61% of the reports lacked gender information, and among the reports that provided gender details, it seemed that males had higher occurrence in AEs than females (43.01% vs. 35.38%). For rocuronium and vecuronium, the occurrence rates of AEs are similar between males and females. From the perspective of age composition, patients were mainly between the ages of 18 and 59 among all NMBAs. Most of these reports were from the United States (31.83%), followed by France (14.42%), and Great Britain (11.21%). Physicians represented the primary source of reports (30.44%).

### Risk analysis at the SOC level

In total, 11,518 AEs were found to be related to NMBAs. Rocuronium was reported the most (7,476 AEs, 64.91%), followed by atracurium (1,702 AEs, 14.78%), vecuronium (1,340 AEs, 11.63%), and cisatracurium (1,000 AEs, 8.68%). These AEs were classified based on SOC of MedDRA, and a total of 27 SOCs were involved. The top five SOCs with the highest number of AEs among the four NMBAs were “general disorders and administration site conditions”, “respiratory, thoracic and mediastinal disorders,” “cardiac disorders,” “injury, poisoning and procedural complications” and “immune system disorders.” The details were described in Supplementary Figure S1.

In order to further understand the risk of NMBA-related adverse events at the SOC level, We compared the number of AEs of the four NMBAs with the number of AEs of the overall population at the same SOC level by disproportionality analysis. The larger the ROR and IC values were, the stronger the signal was (Jiang et al., 2022). As shown in Table 2, there were some differences in SOC involved in the four NMBAs. There were five positive signals for atracurium and rocuronium, six for cisatracurium and vecuronium. All four NMBAs had positive signals at SOC level in “cardiac disorders,” “immune system disorders,” “respiratory, thoracic and mediastinal disorders” and “vascular disorders.” In the aspect of “cardiac disorders,” all four NMBAs exhibited medium signals (++). In terms of “immune system disorders,” the signals of rocuronium were identified as strong signal (+++), atracurium and cisatracurium were medium signals (++), while vecuronium showed a weak signal (+). For “pregnancy, puerperium and perinatal conditions,” atracurium showed a weak signal (+), while the other three NMBAs showed no signal. For “hepatobiliary disorders,” cisatracurium showed a weak signal (+).

**TABLE 2:** Signal strength of NMBA-related AEs at the SOC level.

SOC	N^a^	ROR (95% CI)	IC (IC-2SD)
Atracurium			
Cardiac disorders	195	4.66 (4.01–5.41)	2.08 (1.84)
Immune system disorders	176	10.45 (8.94–12.22)	3.24 (2.95)
Pregnancy, puerperium and perinatal conditions	17	2.28 (1.41–3.67)	1.18 (0.40)
Respiratory, thoracic and mediastinal disorders	272	3.86 (3.39–4.39)	1.77 (1.56)
Vascular disorders	171	5.05 (4.31–5.91)	2.21 (1.95)
Cisatracurium			
Cardiac disorders	117	4.77 (3.93–5.79)	2.11 (1.79)
Hepatobiliary disorders	21	2.32 (1.51–3.58)	1.20 (0.49)
Immune system disorders	99	9.96 (8.09–12.25)	3.18 (2.76)
Investigations	82	1.35 (1.08–1.70)	0.41 (0.07)
Respiratory, thoracic and mediastinal disorders	104	2.35 (1.92–2.88)	1.15 (0.83)
Vascular disorders	76	3.72 (2.94–4.69)	1.81 (1.42)
Rocuronium			
Cardiac disorders	911	5.00 (4.66–5.36)	2.17 (2.07)
Immune system disorders	965	13.45 (12.57–14.39)	3.56 (3.45)
Injury, poisoning and procedural complications	869	1.18 (1.10–1.27)	0.22 (0.11)
Respiratory, thoracic and mediastinal disorders	871	2.68 (2.49–2.87)	1.31 (1.20)
Vascular disorders	532	3.46 (3.17–3.78)	1.72 (1.58)
Vecuronium			
Cardiac disorders	163	5.88 (4.23–5.88)	2.17 (1.90)
Immune system disorders	69	6.27 (3.86–6.27)	2.24 (1.81)
Injury, poisoning and procedural complications	240	2.26 (1.71–2.26)	0.84 (0.63)
Investigations	123	1.84 (1.27–1.84)	0.57 (0.29)
Respiratory, thoracic and mediastinal disorders	150	3.03 (2.16–3.03)	1.25 (0.99)
Vascular disorders	56	2.57 (1.51–2.57)	0.95 (0.53)

^a^ Number of adverse events; NMBAs, Neuromuscular blocking agents; AEs, adverse events; SOC, system organ; ROR, reporting odds ratio; 95%CI, 95% confdence interval; IC, information component; IC-2SD, information component-2, standard deviations.

### Risk analysis at the PT level

**FIGURE 1:**
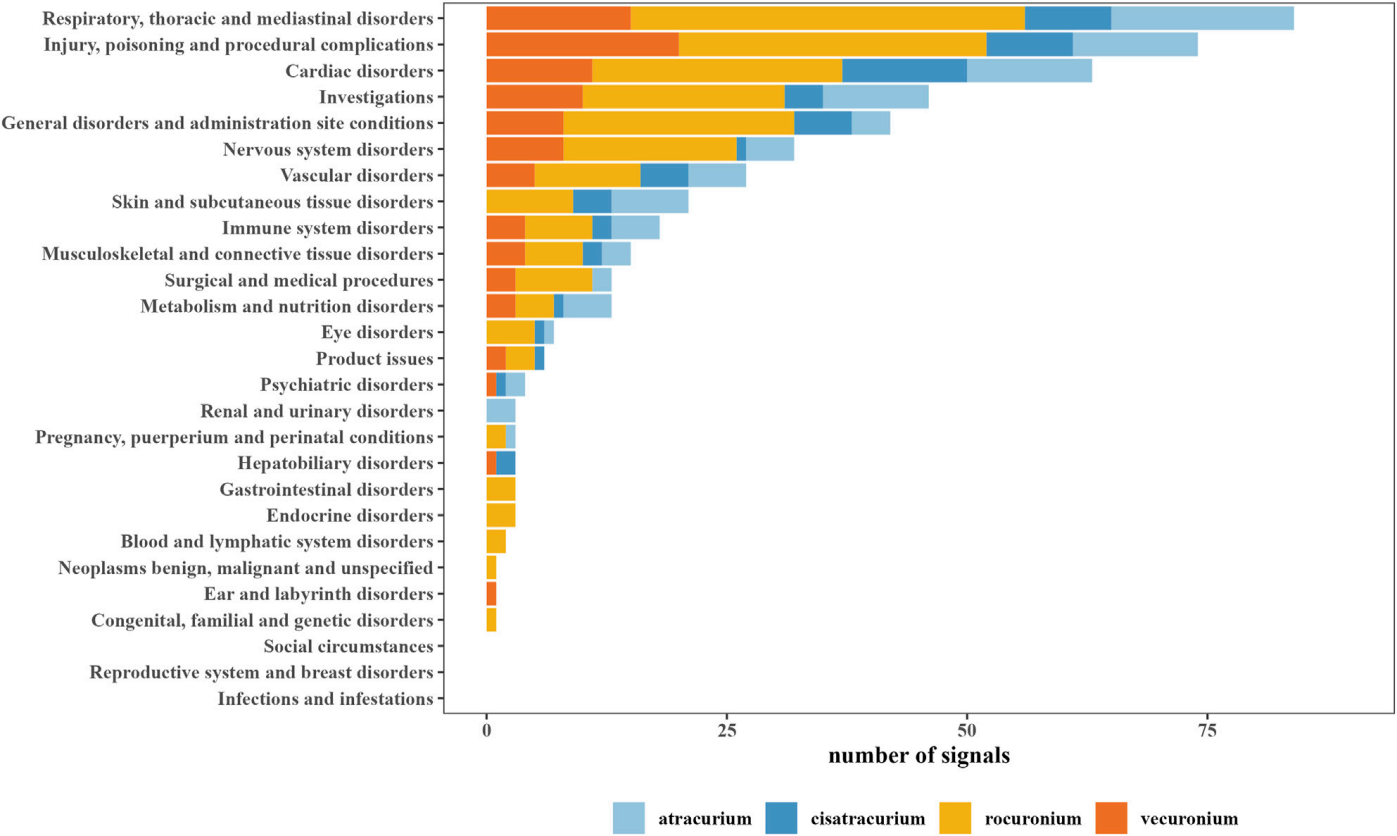
System organ class (SOC) of positive signals related to neuromuscular blocking agents (NMBAs).

Based on the AEs induced by all other drugs in the FAERS database, we further analyzed the PT signals for each NMBA to explore whether there was any association between NMBAs and PTs. According to the criteria of these two algorithms, we identified positive signals for atracurium, cisatracurium, rocuronium, vecuronium as 101, 61, 227, and 96, respectively. The most number of positive signals for the four NMBAs were focused on “respiratory, thoracic and mediastinal disorders,” followed by “injury, poisoning and procedural complications,” “cardiac disorders,” “Investigations,” “general disorders and administration site conditions.” Figure 1 provided an overview of the number of positive signals for the four NMBAs in the corresponding SOC.

The top 20 NMBA-related AEs were listed in Table 3. The PTs were arranged in ascending alphabetical order. Notably, anaphylactic reaction, anaphylactic shock, bronchospasm, bradycardia, cardiac arrest, hypotension, circulatory collapse, tachycardia and respiratory arrest were all identified positive signal for all NMBAs. Comparing the signal intensity of the four NMBAs, we found that the signal intensity of rocuronium was the strongest, while cisatracurium had the characteristic of having fewer signals, low signal strength, and overlapping signals with other NMBAs.

**TABLE 3:** Signal strength of the top 20 NMBA-related AEs at the PT level.

PTs	Atracurium			Cisatracurium			Rocuronium			Vecuronium		
	N^a^	ROR (95% CI)	IC (IC-2SD)	N^a^	ROR (95% CI)	IC (IC-2SD)	N^a^	ROR (95% CI)	IC (IC-2SD)	N^a^	ROR (95% CI)	IC (IC-2SD)
Anaphylactic reaction	81	59.82 (47.85–74.80)	5.83 (4.75)	19	23.15 (14.70–36.46)	4.51 (2.79)	530	92.33 (84.48–100.90)	6.41 (6.06)	23	20.88 (13.82–31.54)	4.36 (2.91)
Anaphylactic shock	66	101.31 (79.18–129.62)	6.60 (4.96)	68	183.24 (143.19–234.48)	7.41 (5.26)	296	104.72 (93.15–117.73)	6.63 (6.05)	24	45.70 (30.51–68.45)	5.49 (3.44)
Blood pressure decreased	12	6.49 (3.68–11.45)	2.69 (1.38)	8	7.37 (3.67–14.77)	2.87 (1.14)	74	9.14 (7.27–11.50)	3.18 (2.70)	13	8.95 (5.18–15.46)	3.15 (1.73)
Bradycardia	27	17.71 (12.11–25.91)	4.12 (2.91)	11	12.22 (6.74–22.13)	3.60 (1.81)	124	18.57 (15.55–22.18)	4.19 (3.74)	28	23.45 (16.13–34.10)	4.55 (3.17)
Bronchospasm	62	156.93 (121.70–202.35)	7.23 (5.11)	16	67.24 (41.02–110.23)	6.05 (3.07)	185	106.40 (91.85–123.25)	6.68 (5.83)	15	46.81 (28.13–77.89)	5.53 (2.87)
Cardiac arrest	25	10.54 (7.10–15.65)	3.38 (2.36)	22	15.91 (10.42–24.27)	3.96 (2.64)	281	27.71 (24.59–31.23)	4.73 (4.43)	27	14.54 (9.93–21.29)	3.83 (2.72)
Circulatory collapse	30	60.63 (42.24–87.02)	5.89 (3.84)	7	23.78 (11.31–50.03)	4.56 (1.60)	105	48.37 (39.87–58.69)	5.57 (4.76)	8	20.26 (10.11–40.61)	4.33 (1.72)
Drug effect decreased	4	2.19 (0.82–5.85)	1.13 (−0.47)	3	2.80 (0.90–8.70)	1.48 (−0.50)	72	9.06 (7.19–11.43)	3.17 (2.68)	12	8.41 (4.77–14.86)	3.06 (1.61)
Drug ineffective	17	0.46 (0.29–0.74)	−1.10 (−1.74)	61	2.96 (2.29–3.84)	1.51 (1.09)	514	3.37 (3.08–3.68)	1.68 (1.54)	72	2.59 (2.04–3.28)	1.32 (0.95)
Dyspnoea	8	0.51 (0.25–1.01)	−0.98 (−1.86)	9	0.97 (0.50–1.87)	−0.04 (−0.95)	52	0.75 (0.57–0.98)	−0.41 (−0.80)	6	0.48 (0.22–1.07)	−1.05 (−2.03)
Erythema	10	1.74 (0.94–3.25)	0.80 (−0.17)	9	2.68 (1.39–5.17)	1.41 (0.27)	69	2.75 (2.17–3.49)	1.45 (1.07)	5	1.11 (0.46–2.66)	0.14 (−1.06)
Hypotension	72	13.32 (10.52–16.86)	3.68 (3.12)	32	9.96 (7.01–14.17)	3.27 (2.43)	259	10.83 (9.57–12.26)	3.39 (3.15)	19	4.33 (2.76–6.82)	2.10 (1.23)
Hypoxia	6	6.33 (2.84–14.11)	2.66 (0.75)	8	14.43 (7.19–28.93)	3.84 (1.56)	68	16.45 (12.96–20.90)	4.02 (3.39)	8	10.74 (5.36–21.53)	3.42 (1.40)
Neuromuscular block prolonged	2	180.93 (45.02–727.11)	7.49 (−0.11)	6	939.84 (418.11–2,112.63)	9.84 (1.69)	139	5,024.88 (4,031.76–6,262.62)	11.47 (6.77)	24	3,007.63 (1977.99–4,573.26)	11.42 (4.03)
Oxygen saturation decreased	25	17.56 (11.83–26.07)	4.11 (2.84)	10	11.89 (6.38–22.18)	3.56 (1.70)	87	13.89 (11.24–17.16)	3.78 (3.27)	9	7.96 (4.13–15.34)	2.98 (1.31)
Paralysis	9	22.06 (11.46–42.49)	4.46 (1.91)	5	20.85 (8.66–50.22)	4.37 (1.09)	48	26.91 (20.25–35.76)	4.74 (3.71)	21	66.15 (42.97–101.84)	6.02 (3.43)
Rash	22	1.91 (1.26–2.92)	0.93 (0.27)	11	1.63 (0.90–2.95)	0.70 (−0.22)	85	1.68 (1.36–2.08)	0.74 (0.42)	5	0.55 (0.23–1.32)	−0.86 (−1.93)
Respiratory arrest	23	27.32 (18.10–41.24)	4.75 (3.10)	7	9.56 (4.55–20.12)	3.25 (1.18)	61	16.43 (12.77–21.15)	4.02 (3.34)	27	41.02 (28.02–60.07)	5.33 (3.51)
Tachycardia	31	12.66 (8,87–18.06)	3.64 (2.68)	9	6.20 (3.21–11.94)	2.62 (1.10)	130	12.09 (10.16–14.38)	3.57 (3.20)	17	8.77 (5.43–14.15)	3.12 (1.92)

^a^ Number of adverse events; NMBAs, Neuromuscular blocking agents; AEs, adverse events; PT, preferred Term.

Comparing the detected positive signals with the AEs labeled in the instructions, 59, 36, 127, 43 unlabeled AEs were identified for atracurium, cisatracurium, rocuronium and vecuronium, respectively. Besides the AEs listed in Table 3, we also found other new AEs (Supplementary Table S1). For example, atracurium was identified to be associated with hyperglycaemia (IC-2SD = 2.64), ventricular fibrillation (IC-2SD = 2.00) and blood creatine phosphokinase increased (IC-2SD = 2.12), all of which showed medium signals (++). For cisatracurium, medium signals included ventricular fibrillation (IC-2SD = 2.15) and rhabdomyolysis (IC-2SD = 1.65), weak signals included hepatocellular injury (IC-2SD = 1.39). For rocuronium, kounis syndrome (IC-2SD = 3.60) was strong positive signal (+++), stress cardiomyopathy (IC-2SD = 2.56), ventricular fibrillation (IC-2SD = 2.72) and rhabdomyolysis (IC-2SD = 1.64) were medium signals (++). For vecuronium, ventricular fibrillation (IC-2SD = 2.26) was identified as medium signals (++), hyperkalaemia (IC-2SD = 1.39) and rhabdomyolysis (IC-2SD = 1.03) were identified as weak signals (+).

### Outcomes of NMBA-related adverse events

**FIGURE 2:**
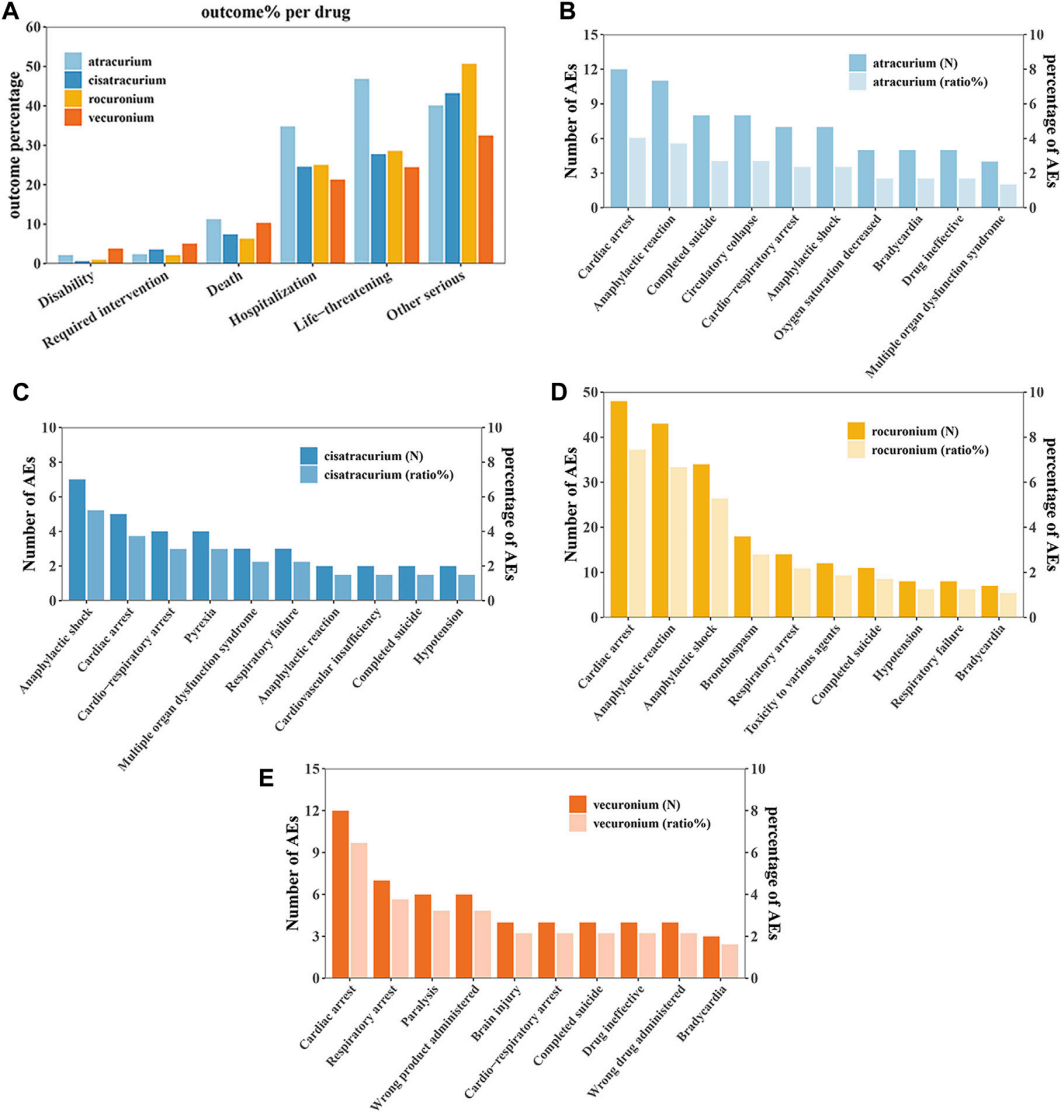
Outcomes of NMBA-related adverse events. **(A)** Outcomes for adverse events associated with Neuromuscular blocking agents (NMBAs). **(B)** The top 10 AEs leading to death for atracurium. **(C)** The top 10 AEs leading to death for cisatracurium. **(D)** The top 10 AEs leading to death for rocuronium. **(E)** The top 10 AEs leading to death for vecuronium.

3,904 patients had experienced serious clinical outcomes, indicating that NMBAs may have potentially dangerous attributes. For outcome, since a patient could experience multiple outcomes, the total sum of percentages may be greater than 100%, but this is not contradictory. As shown in Figure 2A, atracurium had the highest reported rates of death, hospitalization (initial or prolonged) and life-threatening complications, with rates of 11.26%, 34.78%, and 46.84%, respectively, while rocuronium had the lowest percentage of death (6.32%), vecuronium had the lowest percentage of hospitalization (initial or prolonged) and life-threatening (21.31% and 24.47%, respectively). A total of 341 patients reported death outcome, in order to further explore the AEs leading to death, we assessed the mortality caused by different AEs of the four drugs based on the number of deaths reports. Among them, cardiac arrest and anaphylaxis were the main risk factors for death due to atracurium (Figure 2B), cisatracurium (Figure 2C), rocuronium (Figure 2D) and vecuronium (Figure 2E).

## Discussion

Although NMBAs have been used for a long time in clinical practice, there is still a lack of big data research to evaluate post-marketing AEs of NMBAs. As far as we know, this is the first study on the safety of NMBAs based on FAERS. In this study, we used two statistical methods (ROR and BCPNN) to mine the potential AEs signals of NMBAs in the FAERS database. These two methods can validate each other and quantitatively reflect the correlation between target drugs and AEs. The AEs labled in the instructions (such as anaphylaxis, bronchospasm, respiratory arrest and hypotension) were detected in above, indicating that the AEs signal mining based on the FAERS system was reliable and predictable.

At the level of SOC, the large-scale comparison of NMBAs was carried out, and it demonstrated that the four NMBAs all had positive signals in “cardiac disorders,” “immune system disorders,” “respiratory, thoracic and mediastinal disorders,” and “vascular disorders.” Cisatracurium was the only drug associated with “hepatobiliary disorders,” which has not been reported before. Atracurium showed positive signal in “pregnancy, puerperium and perinatal conditions,” the PTs includes neonatal disorder. A study has found that due to the low degree of placental transfer of atracurium, no health issues were observed in the newborns of pregnant women after they were administered atracurium as a muscle relaxant (Flynn et al., 1984). Greco et al., (1995) found that during intrauterine surgery, the use of atracurium on the fetus did not result in side effects related to paralysis at birth and during the 2-year follow-up period. Our results were inconsistent with these studies, and the possible reasons are as follows: First, the current data on the safety of atracurium during pregnancy were limited, and the sample sizes of existing studies were relatively small, whereas our methodology encompassed a larger sample size. Second, AEs related to newborns may be distributed across different PTs/SOCs, which may lead to bias in the results. Consequently, further research, including well-designed clinical trials, is necessary to provide a stronger understanding of the safety of atracurium use in pregnant women and their offspring.

This study excavated signs of AEs that were not labeled in the instructions, such as ventricular fibrillation for the four NMBAs, hyperglycaemia for atracurium, kounis syndrome and stress cardiomyopathy for rocuronium, hepatocellular injury for cisatracurium, hyperkalaemia for vecuronium. Some researchers believe that the mechanism behind rocuronium-induced kounis syndrome involves complex immune responses and allergic processes. Rocuronium triggers the activation of mast cells, which can selectively release mediators such as interleukin-1, serotonin and leukotrienes, thereby promoting coronary artery inflammation (Fagley et al., 2009; Del Val Villanueva et al., 2018). Regarding other new AEs, since there are currently no relevant reports, the mechanisms are unclear, and thus a definitive conclusion cannot be drawn and further validation requires.

Currently, the focus of research on AEs associated with NMBAs has been primarily on IgE-mediated anaphylaxis (Baldo et al., 2009; Petitpain et al., 2018). The majority of reports on the incidence of anaphylaxis originate in Australia (Fisher et al., 1993), France (Laxenaire, 1993), the United Kingdom (Clarke et al., 1993) and New Zealand (Galletly et al., 1985). Anaphylaxis accounted for the largest proportion of NMBAs adverse event reports in the FAERS database. Compared with other NMBAs, our results show that rocuronium has the highest number of reported anaphylaxis, and previous clinical studies had also found that rocuronium were markedly more involved in perioperative anaphylaxis than the other available NMBAs. A regulatory information had already been sent to French anesthetists to communicate about the results of a survey revealing a higher frequency of anaphylaxis with rocuronium than with other NMBAs (Mertes et al., 2003). Sadleir et al., (2013) also highlighted the high incidence of rocuronium induced anaphylaxis in a 10-year survey performed in a specialized diagnostic center. They found that rocuronium was responsible for 56% of cases of NMBA anaphylaxis and had a higher rate of IgE-mediated anaphylaxis compared with vecuronium. Petitpain et al., (2018) recently reported that rocuronium has an anaphylaxis incidence rate 11-fold higher than atracurium and 37-fold higher than cisatracurium, they also found that the annual incidence rates of rocuronium showed increasing trend. However, the debate on the AEs of NMBAs is still controversial. Some reports suggest a higher frequency of AEs involving rocuronium (Sadleir et al., 2013; Petitpain et al., 2018), whereas others consider that the incidence reflect market use (Rose et al., 2001; Watkins, 2001). In the FAERS database, the AEs of rocuronium were much more than other NMBAs. This variation may be due to poor reporting and/or reporting bias, which means that clinicians reported the AEs of rocuronium and ignored other NMBAs adverse events. There is also a possibility that, as reported in most clinical studies, the incidence of rocuronium adverse events is higher than other NMBAs.

NMBAs can affect the cardiovascular system through a variety of mechanisms, such as causing autonomic nerve balance disorders, releasing histamine or causing anaphylaxis (Hameedullah et al., 1997). Individuals receiving NMBAs can be expected to represent critically ill patients. These patients may have higher risk for cardiovascular adverse events as over half of the patients were already receiving vasopressors or inotropic drugs prior to initiation of NMBAs (VanderWeide et al., 2017). It has been reported that rocuronium mainly causes tachycardia and rarely causes bradycardia (Harvey et al., 1999). However, we had detected strong positive signal of bradycardia in rocuronium. In addition, we also found that these four NMBAs were associated with other cardiovascular adverse events, such as hypotension, cardiac arrest, circulatory collapse and ventricular fibrillation. It has been proposed that hypotension resulted by atracurium may be associated with histamine release (Hosking et al., 1988; Murray et al., 2016), and several studies have demonstrated that the combined use of an H_1_ and H_2_ antagonist can attenuate this response (Scott et al., 1985; Hosking et al., 1988; Murray et al., 2016). However, cisatracurium, rocuronium, and vecuronium do not release histamine (Murray et al., 2016), but clinical studies (Hameedullah et al., 1997; Jeong et al., 2010; VanderWeide et al., 2017) and our results have shown that these three drugs are also associated with hypotension, suggesting that the three NMBAs may cause hypotension through other mechanisms.

Bronchospasm is an adverse event associated with NMBAs, which is documented in the instructions or confirmed in previous clinical trials (Woods et al., 1985; O’Callaghan et al., 1986; Heier et al., 2000; Wang et al., 2021). However, fewer cases were reported and the reporting time was earlier. We found that these four NMBAs had strong positive signals of bronchospasm, suggesting a high risk of NMBA-induced bronchospasm. Our study reminds anesthetists to pay attention to bronchospasm caused by NMBAs and avoid serious respiratory complications such as respiratory failure and respiratory arrest.

As shown in the results above, atracurium showed the the highest reported rates of death, hospitalization (initial or prolonged), and life-threatening complications. Besides the histamine release caused by atracurium, we believe that it is also related to the use of its antagonists. Currently, the available antagonists for NMBAs include neostigmine and sugammadex. Neostigmine can be used to reverse any non-depolarizing NMBAs, while sugammadex can only be used to reverse rocuronium and vecuronium (Thilen et al., 2023). Compared with sugammadex, neostigmine cannot reverse deep neuromuscular blockade, which means that if deep blockade occurs after the use of atracurium, neostigmine cannot be given until it returns to shallow blockade. However, if deep neuromuscular blockade occurs after the use of rocuronium or vecuronium, sugammadex can be selected to rapidly reverse it (Thilen et al., 2023).

There is limited data on fatalities specifically attributed to NMBAs in published studies. Cardiac arrest and anaphylaxis have been shown to be the main risk factors for death due to NMBAs. Apart from atracurium, the instructions for the other three NMBAs do not mention cardiac arrest, and there is almost no literature reporting NMBA-related cardiac arrest. Anaphylaxis are likely to cause serious consequences. Reitter et al., (2014) had found that despite aggressive use of epinephrine and fluid therapy, approximately 4% of cases of NMBAs anaphylaxis still result in fatalities. Petitpain et al., (2018) suggested use skin test to avoid anaphylaxis. Taken together, for patients using NMBAs, anesthetists should be vigilant about anaphylaxis and the patient’s cardiac system, maintain surveillance throughout the treatment process to ensure the best outcome.

It is worth noting that our study has some limitations. First, the FAERS database relies on voluntary reporting, which may lead to underreporting or reporting biases. Second, spontaneous reporting systems are usually considered inappropriate for the assessment of adverse drug reaction rates. Third, our study spans nearly 20 years, during which clinical practices, NMBA usage patterns, and reporting practices may have changed, potentially affecting the comparability of AEs among the four NMBAs. Fourth, it is difficult to control confounding factors, such as comorbidities, drug combinations, and other factors that may affect AEs. Therefore, establishing clear causal relationship between NMBAs and AEs is restricted, and the disproportionality analysis can only provide statistical associations. Our research is an important step in identifying potential safety signals, and further experimental studies are needed to validate these results. Despite these limitations, the FAERS database remains a valuable resource for pharmacovigilance and identifying potential safety concerns. Several advantages can be identified. We utilized a large-scale publicly accessible pharmacovigilance database that accepts reports from various locations globally, thus supporting the generalizability of the results (Raschi et al., 2022; Cecco et al., 2024) and helping accumulate knowledge about the safety of NMBAs in an unselected populations, which is still a topic lacking research. In summary, potential AEs signals associated with NMBAs were systematically identified, which can provide valuable evidence for further research and clinical use.

## Conclusion

We reviewed the safety profiles of atracurium, cisatracurium, rocuronium, and vecuronium based on AEs submitted to the FAERS database from the first quarter of 2004 to the third quarter of 2023. According to the analysis results, AEs of NMBAs occurred in multiple organs and tissues, including the cardiac, immune, respiratory and vascular systems. NMBAs have different safety profiles, which may lead to serious adverse events, resulting in hospitalization or death. In order to ensure the safety of patients and reduce the risk of adverse events, anesthetists should be aware of these differences and adjust treatment regimens according to different patients. Although several post-marketing safety signals that were unlabled in the instructions were found, it is necessary to conduct prospective clinical trials to confirm these findings.

## Data Availability

The original contributions presented in the study are included in the article/Supplementary Material, further inquiries can be directed to the corresponding author.
