# Supplementary material for: Adverse events related to neuromuscular blocking agents: a disproportionality analysis of the FDA adverse event reporting system
Source: Front Pharmacol. 2024 Jul 24;15:1403988. doi: 10.3389/fphar.2024.1403988 (PMC11303309; doi:10.3389/fphar.2024.1403988)
Supplement: Supplementary file 2 [file Image1.pdf]

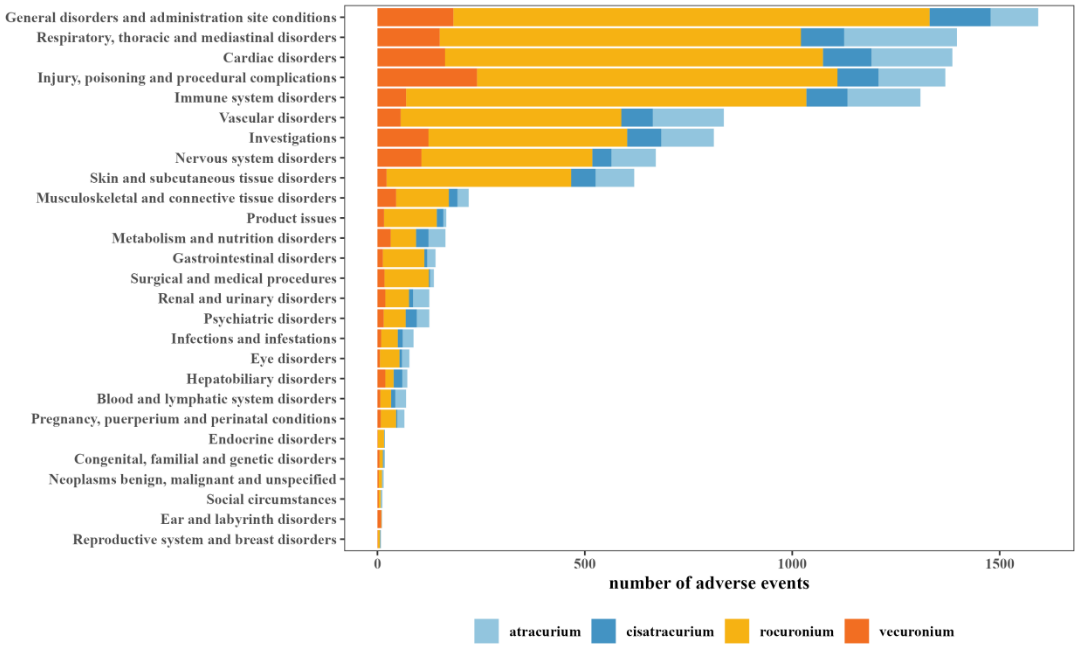

SUPPLEMENTARY FIGURE S1

System organ class (SOC) of adverse events related to neuromuscular blocking agents (NMBAs).
